# Gut microbiota changes in postmenopausal women with low bone density linked to serum amino acid metabolism

**DOI:** 10.3389/fcimb.2025.1627519

**Published:** 2025-07-09

**Authors:** Adriana Becerra-Cervera, Rogelio F. Jiménez-Ortega, Diana I. Aparicio-Bautista, Berenice Palacios-González, Marcela Vela-Amieva, Isabel Ibarra-González, Samuel Canizales-Quinteros, Jorge Salmerón, Berenice Rivera-Paredez, Rafael Velázquez-Cruz

**Affiliations:** ^1^ Laboratorio de Genómica del Metabolismo Óseo, Instituto Nacional de Medicina Genómica (INMEGEN), Mexico City, Mexico; ^2^ Secretaría de Ciencia, Humanidades, Tecnología e Innovación (SECIHTI), Mexico City, Mexico; ^3^ Clínica Integral Universitaria (CIU), Universidad Estatal del Valle de Ecatepec (UNEVE), Ecatepec de Morelos, Estado de Mexico, Mexico; ^4^ Departamento de Medicina Genómica, Instituto Nacional de Rehabilitación (INR), Mexico City, Mexico; ^5^ Laboratorio de Genómica del Envejecimiento del Instituto Nacional de Medicina Genómica (INMEGEN), en el Centro de Investigación sobre Envejecimiento (CIE-CINVESTAV Sur), Mexico City, Mexico; ^6^ Laboratorio de Errores Innatos del Metabolismo y Tamiz, Instituto Nacional de Pediatría, Mexico City, Mexico; ^7^ Unidad de Genética de la Nutrición, Instituto de Investigaciones Biomédicas, Universidad Nacional Autónoma de México (UNAM), Mexico City, Mexico; ^8^ Unidad de Genómica de Poblaciones Aplicada a la Salud, Departamento de Biología, Facultad de Química-Universidad Nacional Autónoma de México (UNAM)/Instituto Nacional de Medicina Genómica, Mexico City, Mexico; ^9^ Centro de Investigación en Políticas, Población y Salud (CIPPS) de la Facultad de Medicina de la Universidad Nacional Autónoma de México (UNAM), Mexico City, Mexico

**Keywords:** bone mineral density, gut microbiota, postmenopausal women, amino acids, bone health

## Abstract

**Background:**

The gut microbiota (GM) has been linked to changes in bone mineral density (BMD), potentially contributing to the development of osteopenia or osteoporosis. Although the relationship between specific bacterial taxa and bone remodeling has been documented in various populations, data on the Mexican population remain limited. This study aimed to analyze the changes in the taxonomic composition of GM associated with low BMD (osteopenia/osteoporosis) and explore potential mechanisms influencing bone metabolism in Mexican postmenopausal women.

**Methods:**

BMD was measured by dual-energy X-ray absorptiometry. GM composition was analyzed through 16S rRNA amplicon sequencing in Mexican postmenopausal women. Metabolic functions were predicted using PICRUSt2 based on KEGG pathways at hierarchy level 3. Serum amino acid (AA) concentrations were measured in a sub-sample using tandem mass spectrometry.

**Results:**

Our findings indicated that beta diversity significantly differed among BMD groups (p<0.05). Women with low BMD showed higher relative abundances of *Bacteroides*, *Parabacteroides*, *Barnesiella*, *Odoribacter*, *Sutterella*, *Butyricimonas*, *Coprobacter*, *Angelakisiella*, and *Oscillibacter*. Functional prediction revealed differences in alanine, valine, leucine, and methionine-related metabolic pathways. These findings were supported by lower serum concentrations of valine, leucine, and methionine in the low BMD group compared to the normal BMD group (p<0.05).

**Conclusion:**

This study provides evidence of the relationship between GM composition and AA concentrations with changes in BMD. These findings highlight promising areas for the development of potential therapeutic interventions

## 1 Introduction

Osteoporosis (OP) is a skeletal and systemic disorder characterized by progressive loss of bone mineral density (BMD), microarchitectural deterioration, and decreased bone strength, leading to risk of fractures (Ensrud and Crandall, 2024). Multiple factors influence bone health include nutritional factors, genetics, metabolic status, and environmental exposures. In recent years, increasing evidence has revealed that gut microbiota (GM) composition plays a pivotal role in the development and progression of OP.

GM promotes the absorption of minerals such as calcium, magnesium, and phosphorus, thereby increasing the BMD through their involvement in the bile acid metabolism and vitamin B and K biosynthesis (Rodríguez et al., 2013; Clarke et al., 2014). Clinical studies have identified distinct taxonomic shifts in osteoporotic patients compared to healthy controls (Xu et al., 2020; Qin et al., 2021; Wang et al., 2022, 2023a). Evidence demonstrates significant taxonomic differences between the GM of osteoporotic patients and healthy controls. For instance, a higher abundance of Enterobacteriaceae members (e.g., Klebsiella, Citrobacter), a reduced Firmicutes/Bacteroidetes ratio and depletion of beneficial taxa such as *Bifidobacterium*, Lachnospiraceae and *Blautia* have been associated with bone loss (Li et al., 2019b; Lyu et al., 2023).

Moreover, functional pathways prediction has revealed depletion of the short-chain fatty acid biosynthesis pathway and increased activity in bacterial peptidase pathway (Ling et al., 2021; Akinsuyi and Roesch, 2023). Several studies have demonstrated distinct amino acids (AAs) metabolic signatures associated with enhanced BMD and reduced fracture incidence (Su et al., 2019; Grahnemo et al., 2023). A Mendelian randomization study and monozygotic twin research have demonstrated that AAs exert osteogenic effects independently of genetic factors (Jennings et al., 2016). Despite these advances, most studies have been conducted in Chinese and European populations (Akinsuyi and Roesch, 2023), with limited data from Latin America. This evidence points out the need to understand the bidirectional nature of host-microbiota-AA interactions in bone homeostasis. Therefore, this study explores the interactions between GM composition and serum metabolites in Mexican postmenopausal women associated with BMD status.

## 2 Materials and methods

### 2.1 Study population

For this analysis, data from the Health Workers Cohort Study (HWCS), collected between 2016 and 2019, were used, which included fecal samples from employees of the Mexican Institute of Social Security (IMSS) and their relatives residing in the urban areas of Central Mexico. Details of the study design and methods have been published previously (Denova-Gutiérrez et al., 2016). Participants with malnutrition, renal damage, or previous report of hormone administration were excluded. Sociodemographic and lifestyle characteristics, as well as detailed medical history, were obtained using a self-administered questionnaire. This study was approved by both the IMSS (no. 12CEI 09 006 14, 17 May 2016) and the National Institute of Genomic Medicine (INMEGEN as per its Spanish acronym) (314-CEI 2018/13, 6 March 2018, and CEI 2023/25, 19 June 2023). All participants gave written informed consent, and all procedures were performed in accordance with the Declaration of Helsinki (13/LO/0078). T-score and Z-score in total hip BMD (TH), lumbar spine (L1-4, LS), and femoral neck (FN) were measured using a Lunar DPX NT dual x-ray absorptiometry (DXA) instrument (Lunar Radiation Corp., Madison WI). The study cohort consisted of 535 participants, of which 20 samples were excluded due to incomplete data and 23 samples were discarded because of insufficient sequencing reads. Participants were stratified into two groups based on their TH-BMD: normal-BMD (T-scores between −1.0 and +1) and low-BMD, which included women with osteopenia (T-scores from −1.0 to −2.5) as well as those diagnosed with osteoporosis (OP) (T-scores below −2.5), in accordance with the World Health Organization’s (WHO) criteria.

### 2.2 DNA extraction and 16S rRNA sequencing

Total DNA was extracted using a QIAamp^®^ DNA Stool Minikit or Power Fecal Pro Kit (QIAGEN, Hilden, Germany), following the manufacturer’s instructions. The DNA concentration and purity were determined using spectrophotometry (Nanodrop 2000c; Thermo Scientific, Wilmington, DE, USA).

The 16S rRNA gene V4 hypervariable region was sequenced using the “Earth Microbiome Project” primers 515F and 806R. The libraries were sequenced on the Illumina MiSeq 2 × 250 platform (Illumina, San Diego, CA, USA) at the Sequencing Unit of the INMEGEN. Further details are described in our previous study (López-Montoya et al., 2023).

### 2.3 Sequence data processing

Raw fastq files were analyzed using the QIIME2 (Quantitative Insights Into Microbial Ecology 2) pipeline. The data were processed to remove adapters, sequences containing barcode mismatches, or low-quality reads (Phred values < 30) using the DADA2 (v1.20.0). Reads were trimmed at 30 bp, and lengths below 220 bp were discarded. The reads were denoised to group into the Amplicon Sequence Variants (ASVs), and chimeric sequences were removed using the “consensus” method. Taxonomic classification was assigned using the SILVA v138–99 reference database. The ASV’s were aligned with the MAFFT algorithm, and a phylogeny tree was built with the FastTree algorithm. All artifacts (ASV table, taxonomy, and tree) and metadata files were imported in R using the qiime2R package (v0.99.34) to generate a phyloseq object. Sequences associated with chloroplast and mitochondria were filtered out. Samples with <10,000 sequence reads were excluded from the study. The random selection of reads for each sample was standardized by rarefaction at 10,584 high-quality read depth.

### 2.4 Bioinformatic analysis

Data analyses were performed in the R environment (v4.2.3). Alpha diversity metrics (observed ASV, Chao1, Shannon, and Simpson) were estimated by the plot_richness function. The diversity among samples (beta diversity) was calculated by Unifrac (unweighted and weighted and principal coordinates analysis (PCoA) was visualized in a two-dimensional component using plot_ordination function. Bar graphs were generated with the relative abundance data by phylum, family, and genera, averaging abundances by groups. The Firmicutes/Bacteroides ratio was measured to indicate gut microbiome dysbiosis. The heat trees analysis was used to examine the differences between age-related BMD and bacterial community composition. The hierarchical structure of taxonomic classifications was quantitatively (using the median abundance) and statistically (using the non-parametric Wilcoxon Rank Sum test) quantified using package metacoder (v. 0.3.5).

The inference of metabolic pathways was predicted with KEGG Orthology (KO) using level three information by Reconstruction of Unobserved States 2 (PICRUST2 v.2.1.3-b). Functional enrichment analysis of differentially abundant gene families was subsequently carried out using Statistical Analysis of Metagenomic Profiles (STAMP) software. Welch’s t-test was the default setting for two-group comparisons and p-values were corrected for multiple testing using the Bonferroni method.

### 2.5 Targeted metabolomics analysis

Concentrations of forty serum metabolites acyl-carnitines, free carnitine, and amino acids were measured in a subsample of women with available microbiota data (n=301) using the approach of targeted metabolomics by electrospray tandem mass spectrometry (Quattro Micro API tandem MS, Waters Inc., Milford, MA, USA). Metabolite levels in serum were analyzed using the commercial kit (NeoBase Non-derivatized MS/MS Kit, Perkin Elmer, Waltham, MA, USA), as previously described (Palacios-González et al., 2020).

Briefly, 20 µL of serum from the postmenopausal women included in the study were poured onto filter paper cards (Whatman 903, Dassel, Germany) and dried at room temperature. The spot was cut into 2-mm circles and placed in a 96-well plate. The extraction solution was added to the plate and incubated for 30 min at 30°C at 650× g. Finally, 10 µL of each sample were injected into the flow at 4-min intervals. The Micromass Quattro equipment (Waters Inc., Milford, MA, USA) was used coupled to an ESI source in positive mode. Nitrogen gas was used for desolvation and nebulization, and argon as the collision gas.

### 2.6 Statistical analysis

Data on body measurements, biochemical tests, and BMD measurements are presented as the median and interquartile range (P25-P75). Differential abundance analysis at all taxonomic levels was performed with the linear discriminant analysis effect size (LEfSe v1.0) via microbiomeMarker R package, an LDA score of at least 1.5, and a p < 0.05 to determine what was statistically significant. A permutational multivariate analysis of variance (PERMANOVA) test was used to determine differences between groups using the adonis2 function. Differential analyses between groups were performed with the Wilcoxon test or chi2 test, with p < 0.05 indicating a significant difference. All plots were generated using the ggplot2 package.

A Spearman correlation heatmap was created to assess possible correlations between gut microbiota and clinical data. The correlations between different AA concentrations and the bacterial taxa were calculated using the Spearman test on R software (v3.3.1) using “vegan” and “ggcor” packages. A p < 0.05 was considered to indicate statistical significance.

## 3 Results

### 3.1 Characteristics of the study population

As shown in **Table 1**, the demographic and clinical characteristics of 535 women were analyzed. The median age was 60 years (P25-P75, 54-67), with a prevalence of overweight and obesity of 41.5% and 28.4%, respectively. Based on T-scores criteria, participants were classified into normal-BMD (n=344) and low-BMD (n=191) groups. Significant differences were observed in BMD values at the total hip, femoral neck, and lumbar spine. Furthermore, BMI, body fat proportion, waist circumference, uric acid, ALT, and calcium supplementation differed significantly between the groups.

**Table 1:** Demographic and clinical characteristics of selected postmenopausal women of the Health Workers Cohort Study.

Variable	Total	Normal-BMD	Low-BMD	P value
	n=535	n=344	n=191	
Age^a^,years	60.0 (54.0-67.0)	57.0 (51.0-64.0)	63.0 (59.0-71.0)	<0.001
Age categories, n (%)				
<47 years	43 (8.0)	40 (11.6)	3 (1.6)	
47–60 years	237 (44.3)	176 (51.2)	61 (31.9)	
60–65 years	94 (17.6)	54 (15.7)	40(21.0)	
>65 years	161 (30.1)	74 (21.5)	87(45.5)	
BMI^a^, kg/m^2^	27.2 (24.4-30.9)	28.3 (25.8-32.0)	25.0 (22.8-28.1)	<0.001
Nutritional status, n(%)				
Normal	161 (30.1)	66 (19.2)	95 (49.7)	<0.001
Overweight	222 (41.5)	152 (44.2)	70 (36.6)	0.09
Obesity	152 (28.4)	126 (36.6)	26 (13.6)	<0.001
Body fat proportion^a^	45.5 (41.4-49.8)	46.4 (42.7-50.3)	44.0 (39.4-47.3)	<0.001
Waist circumference^a^, cm	92.0 (85.0-99.0)	94.0 (87.5-101.0)	87.0 (82.0-95.0)	<0.001
Uric acid ^a^, mg/dl	5.0 (4.2-5.8)	5.1 (4.3-6.0)	4.7 (4.1-5.5)	0.0009
ALT ^a^, U/L	26.0 (19.0-36.0)	28.0 (20.0-40.0)	23.0 (18.0-30.0)	<0.001
AST ^a^, U/L	25.0 (22.0-32.0)	26.0 (22.0-33.0)	25.0 (22.0-31.0)	0.5443
Total cholesterol ^a^, mg/dl	202.0 (174.0-232.0)	201.5 (172.0-232.0)	202.0 (176.0-234.0)	0.5726
Triglycerides ^a^, mg/dl	142.0 (107.0-197.0)	145.5 (114.0-202.0)	136.0 (96.0-191.0)	0.0501
HDL ^a^, mg/dl	52.8 (44.0-61.5)	52.1 (43.4-60.1)	53.5 (46.4-64.4)	0.165
LDL ^a^, mg/dl	116.0 (93.9-141.9)	115.8 (93.2-143.8)	116.8 (95.8-139.3)	0.643
Total hip BMD^a^, g/cm^2^	0.936 (0.827-1.030)	1.003 (0.945-1.079)	0.798 (0.738-0.835)	<0.001
Lumbar spine BMD^a^, g/cm^2^	1.027 (0.926-1.137)	1.093 (1.002-1.180)	0.925 (0.849-1.012)	<0.001
Femoral neck BMD^a^, g/cm^2^	0.890 (0.784-0.983)	0.955 (0.889-1.022)	0.757 (0.712-0.809)	<0.001
T-score ^a^, total hip	-0.572 (-1.432,0.176)	-0.041 (-0.497,0.566)	-1.661 (-2.141,-1.367)	<0.001
T-score^a^, lumbar spine	-1.416 (-2.277,-0.604)	-0.920 (-1.696,-0.206)	-2.299 (-2.882,-1.563)	<0.001
T-score^a^, femoral neck	-1.064 (-1.821,-0.394)	-0.599 (-1.069,-0.115)	-2.019 (-2.327,-1.644)	<0.001
Vitamin D intake^a^, UI/day	106.9 (64.7-156.8)	108.5 (61.8-155.1)	104.1 (69.0-157.8)	0.8378
Calcium intake^a^, mg/day	793.0 (575.9-1120.3)	780.8 (574.9-1105.1)	799.8 (584.9-1124.1)	0.4175
Calcium supplementation, n(%)	97 (18.1)	45 (13.1)	52 (27.2)	<0.001
Vitamin D supplementation, n(%)	51 (9.5)	28 (8.1)	23 (12.0)	0.1409
Missing	59 (11.0)	36 (10.5)	23 (12.0)	0.577

### 3.2 Gut microbiota characterization

**Figure 1:**
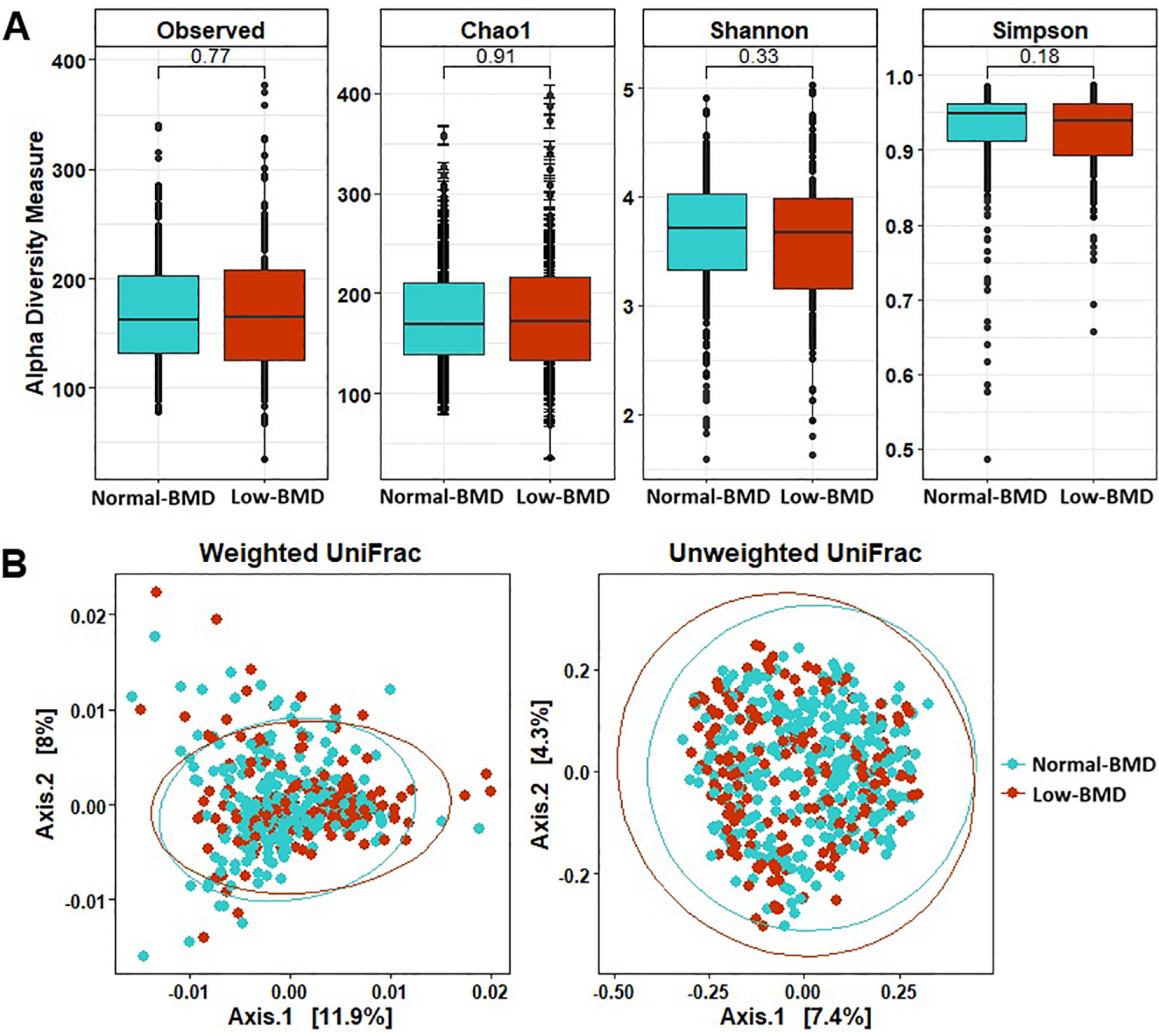
Alpha and beta diversity measures in microbiota structure between in low-BMD and normal-BMD groups. **(A)** Alpha diversity measures with the most common indices. **(B)** PCoA plots showing the beta diversity with unweighted and weighted UniFrac measures. Blue: Normal -BMD samples, red: Low-BMD. Box plots show median, as well as lower and upper quartiles. Each dot represents an individual sample.

A total of 23,698,249 high-quality paired sequences were obtained from fecal samples, with an average sequencing depth of 52,662 (range 10,589 –149,109), which were clustered into 7902 ASVs. Phylogenetic characterization was associated with 13 phyla, 98 families, and 305 genera in the data set. There were no significant differences in microbial richness and alpha diversities between BMD groups (p > 0.05). However, principal-coordinate analysis (PCoA) unweighted and weighted UniFrac distance for beta diversity showed significant differences (p < 0.01) of the bacterial communities between groups (**Figure 1**).

Taxonomic analysis of the bacterial community showed that the dominant phyla in both study groups were Firmicutes (70.8% in the normal and 65% in the low-BMD groups), followed by Bacteroidota (20.3% and 25.4% in the normal and low BMD groups, respectively) (**Supplementary Figure 1**). The predominant families were Lachnospiraceae (36.6% of the normal and 32.9% of the low BMD groups), Bacteroidaceae (10.4% of the normal and 14.7% of the low BMD groups) and Ruminococcaceae (12.1% and 10.1%, in the normal and low BMD groups, respectively). At genus level, *Bacteroides* (10.4% and 14.7% in the normal and low BMD groups, respectively), and *Prevotella* (6.7% and 6.1%) were the most abundant taxa in the normal and low BMD groups, respectively (**Supplementary Figure 1**).

### 3.3 Differences in the abundance of gut bacteria between BMD groups

**Figure 2:**
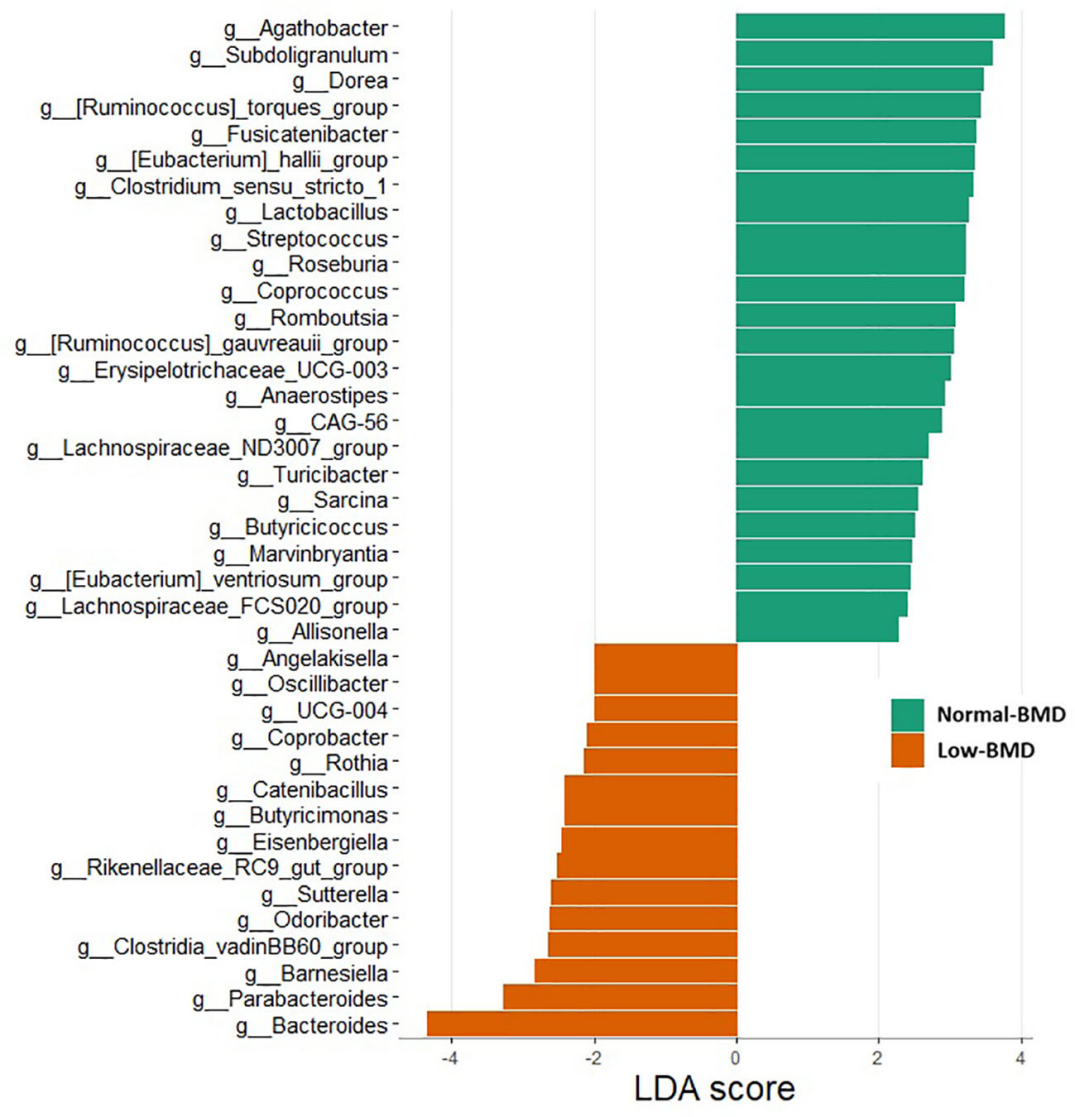
The linear discriminant analysis (LDA) effect size (LEfSe) of genera between normal-BMD and low-BMD women. Genus with LDA score > 1.5 and p < 0.05 are shown.

**Figure 2** shows that 15 and 23 genera were enriched in the low-BMD and normal-BMD, respectively. Several genera, including *Bacteroides*, *Parabacteroides*, *Barnesiella*, *Odoribacter*, *Sutterella, Butyricimonas*, *Coprobacter*, *Angelakisiella*, *and Oscillibacter* were significantly more abundant in the low compared to the normal-BMD group (p < 0.05) (**Figure 2**; **Supplementary Figures 2, 3**). In contrast, *Agathobacter*, *Subdogranulum*, and *Dorea*, belonging to Firmicutes phylum, were enriched in the normal-BMD group. The Firmicutes/Bacteroidota ratio was significantly decreased in the low-BMD group (p=0.013) (**Supplementary Figure 4**).

**Figure 3:**
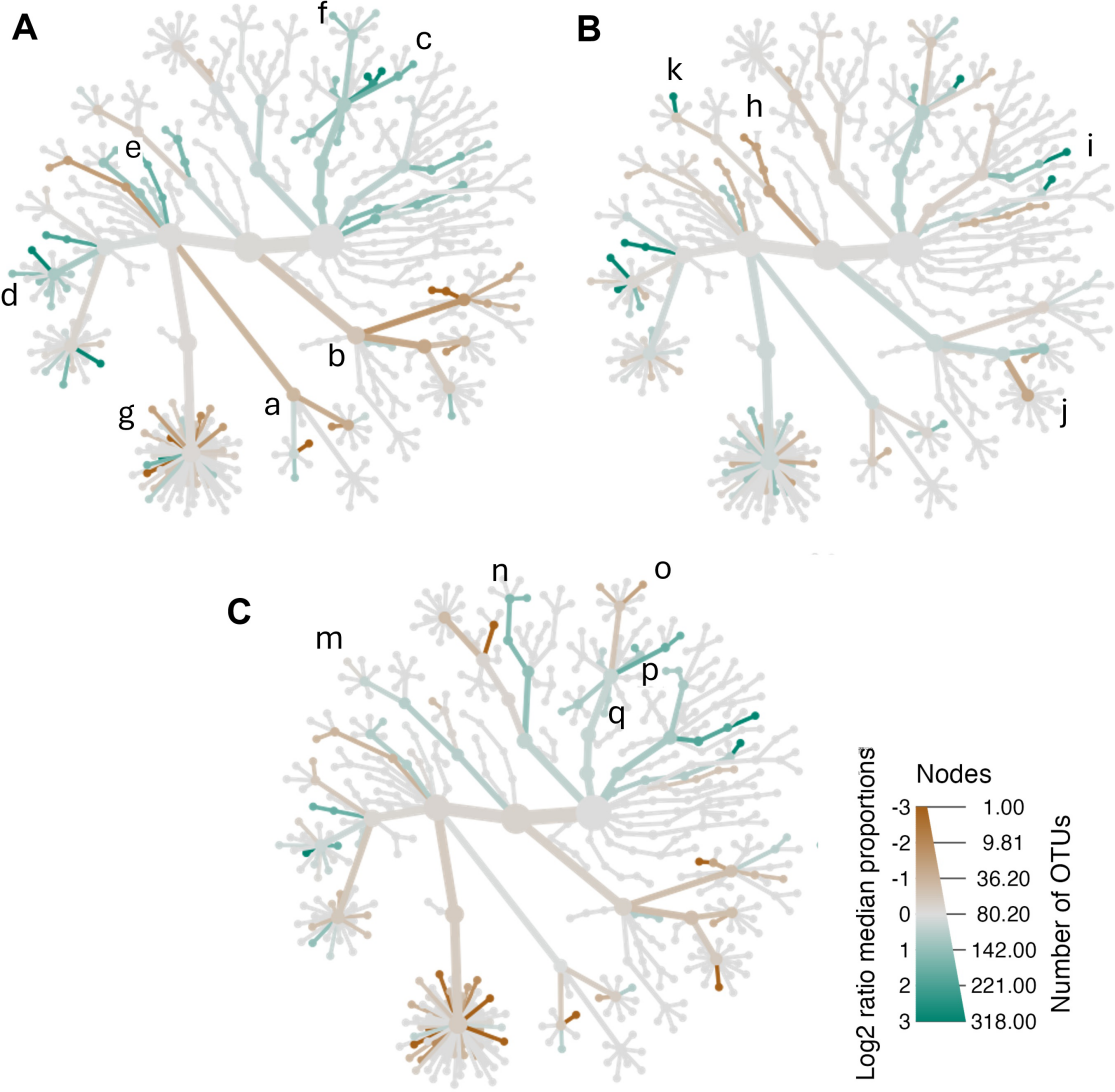
Effects of age-related BMD on bacterial community composition. Heat tree for pair-wise comparison of BMD status, divided by age, **(A)** middle-age women (ages 47–60 years), **(B)** middle-old women (60–65 years), and **(C)** old women (>65 years). The color of each taxon indicates the log-2 ratio of proportions observed in each condition. Taxa colored green are enriched in the low-BMD group and those colored brown are enriched in the normal-BMD group. Peptostreptococcales-Tissierellaes (a) colored brown were enriched in the normal-BMD group; meanwhile, the Barnesiella and Oscillospiracae (Oscillibacter, Flavonifactor) group colored green were enriched in the low-BMD group. a: Peptostreptococcales-Tissierellaes, b: Bacilli (*Enterococcus*, *Lactobacillus*), c: *Barnesiella*, d: Oscillospiracae (*Oscillibacter*, *Flavonifactor*), e: *Clostridia_UCG-014*, f: Prevotellaceae (*Prevotella*), g: Lachnospiraceae (*Agathobacter*, *Marvinbryantia*), h: Negativicutes (*Phascolarctobacterium*), i: *Sutterella*, j: Erysipelotrichaceae (*Holdemania*), k: *Veillonella*, m: Veilloneaceae, n: *Bifidobacterium*, o: *Paraprevotella*, p: *Escherichia-Shigella*, q: *Bacteroides*. For more details consult the heat tree base in **Supplementary Figure 5**; **Supplementary Table 1**. Only those taxa that were statistically significant using the Wilcox rank sum test.

To explore potential age-related microbiota differences, women were divided into three groups: youngest-old (47–60 years), middle-old (61–65 years), and oldest-old (> 65 years). Oscillospiracae (*Oscillibacter*, *Flavonifactor*), *Clostridia_UCG-014*, *Barnesiella*, Prevotellaceae clade (Prevotella) were enriched in youngest-old low-BMD compared to normal BMD (**Figure 3A**); meanwhile, Peptostreptococcales-Tissierellaes clade, Bacilli (*Enterococcus*, *Lactobacillus*) and Lachnospiraceae (*Agathobacter*, *Marvinbryantia*) were enriched in the normal-BMD group (**Figure 3A**). While *Veillonella*, *Paraprevotella*, and *Oscillibacter* were enriched in middle-old women with low-BMD when compared to women of the same age with normal-BMD (**Figure 3B**). The taxa composition of low-BMD in oldest-old showed that *Bifidobacterium*, *Escherichia-Shigella*, as well as Veillonellaceae and Gammaproteobacteria clades were enriched in comparison with normal-BMD of the same age (**Figure 3C**; **Supplementary Figure 5**; **Supplementary Table 1**). In line, *Bacteroides* and *Sutterella* were significantly enriched in low-BMD between aged groups.

### 3.4 Association between serum metabolites and BMD status

**Figure 4:**
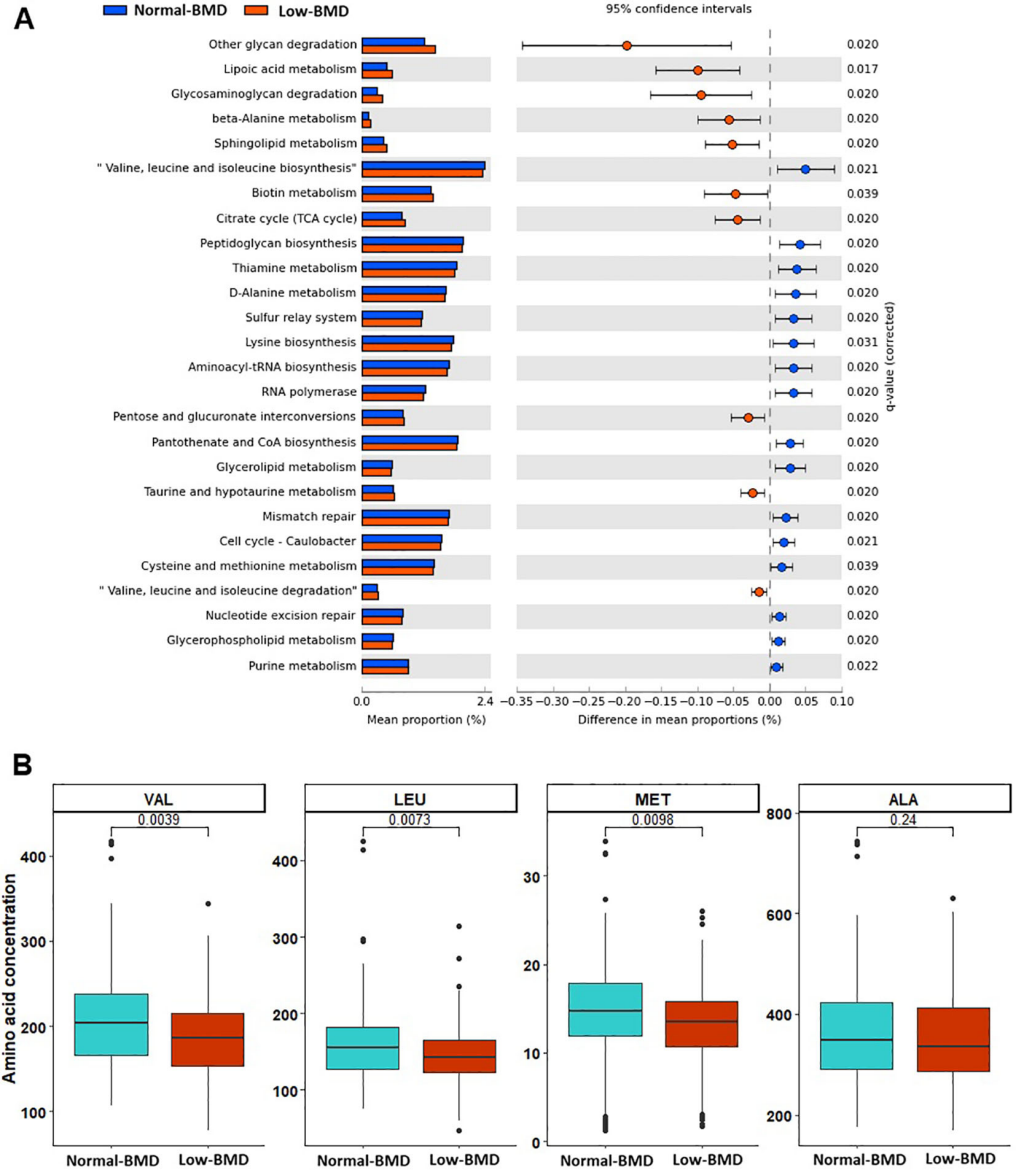
Enrichment analysis pathways and serum amino acid concentrations between in low-BMD and normal-BMD groups. **(A)** Predicted differential KEGG pathways in low-BMD and normal-BMD groups. The extended error bar plot shows significantly differential KEGG pathways predicted using PICRUSt2 analysis and visualized using the STAMP software. Only p values of <0.05 based on Welch’s test are shown. **(B)** The box plot shows the serum amino acid concentrations with significantly different concentrations between groups.

A total of 26 metabolic pathways were enriched between low-BMD and normal-BMD groups. The low-BMD group showed enrichment in pathways associated with lipoic acid metabolism, valine, leucine, and isoleucine degradation, ubiquinone and other terpenoid-quinone biosynthesis, beta-alanine, taurine, hypotaurine, phosphonate, phosphinate, and biotin metabolism. In contrast, the microbiota of normal-BMD had pathways involved in pantothenate and CoA biosynthesis, valine, leucine, and isoleucine biosynthesis, thiamine, glycerolipid, lysine, cysteine, and methionine metabolism. Targeted metabolomic analyses revealed significantly lower valine, leucine and methionine concentrations in women with low BMD compared to those with normal-BMD (**Figure 4**).

### 3.5 Correlation analysis of genera bacteria abundance, BMD measurements and amino acid concentrations

**Figure 5:**
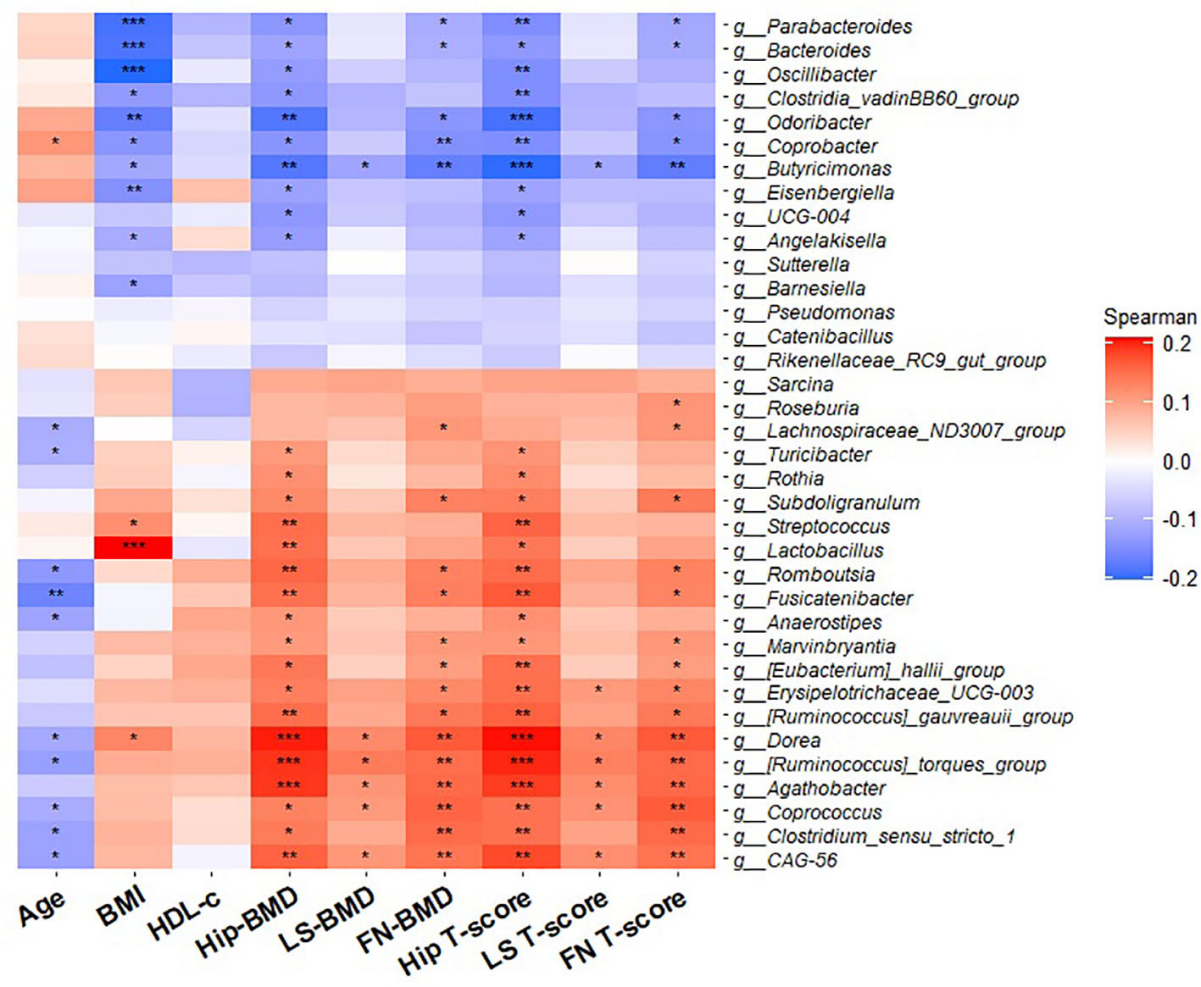
Heatmap of partial Spearman’s rank correlation analysis between differential bacterial genera and clinical characteristics. Red squares indicate positive correlations, and blue squares indicate negative correlations. *Bacteroides* genus abundance was negatively correlated with BMI, Hip-BMD, FN-BMD, Hip T-score and FN T-score. *Coprococcus* genus abundance was negatively correlated with age and in a positive manner with BMD (Hip, LS, and FN) and T-score (Hip, LS and FN). BMD: bone mineral density: HDL-C: high-cholesterol lipoprotein cholesterol; FN, femoral neck; LS, lumbar spine; BMI: body mass index. *p < 0.05, **p < 0.01, and ***p < 0.001.

Five genera, including *Dorea*, Ruminococcus torques group, *Agathobacter*, *Coprococcus*, and CAG-56 showed a positive association with BMD at three specific sites (HT, LS and FN) BMD and T-score (p < 0.05). In contrast, a negative correlation was observed between two sites, hip and FN BMD and T-score with the *Bacteroides*, *Parabacteroides*, *Odoribacter*, *Coprobacter*, and *Butyricimonas* genera. Several genera including Lachnospiraceae ND3007 group, *Turicibacter, Romboutsia, Fusicatenibacter*, *Anaerostipes, Dorea*, *Ruminococcus torques group*, *Coprococcus*, *Clostridium sensu stricto 1*, and CAG-56 were negatively correlated with age (p < 0.05) (**Figure 5**). In total, 13 genera showed a significant correlation with BMI.

Correlation analysis between GM and AA concentrations revealed that genera such as *Subdoligranulum*, *Agathobacter*, Lachnospiraceae_UCG-010, and Lachnospiraceae NK4A136, were positively correlated with serum leucine or valine (**Supplementary Figure 6**). While, *Eubacterium siraeum*, RF39, UCG-005, and Muribaculaceae were negatively correlated with these amino acids. Methionine showed positive associations with *Allisonella* and *Hodelmania* but negative association with RF39 and *Eubacterium siraeum*. Furthermore, alanine was negatively correlated with Oscillospiraceae, while valine was positively correlated with the Bacteroidaceae family (**Supplementary Figure 7**).

## 4 Discussion

This is the first large-scale study analyzing the influence of GM diversity, functional pathways, and metabolomics on bone metabolism in Mexican postmenopausal women with low BMD. Our findings revealed a higher abundance of the genera *Bacteroides*, *Parabacteroides*, *Barnesiella*, *Odoribacter*, *Sutterella*, *Butyricimonas*, *Coprobacter, Angelakisiella*, and *Oscillibacter* associated with low-BMD. Consistent with our findings, previous studies have reported an increased abundance of *Bacteroides* abundance in Asian and Chinese postmenopausal women with low BMD (osteopenia (OS) and osteoporosis (OP) at the femoral neck (FN) or lumbar spine (LS) (Wang et al., 2021; Wei et al., 2021b; Akinsuyi and Roesch, 2023). However, contradictory results have been reported in Chinese individuals with decreased BMD (Ozaki et al., 2021) and postmenopausal women with fractures (Yan et al., 2024). *Parabacteroides* spp. have been found enriched in postmenopausal women with OS and OP across multiple Chinese cohorts (Wang et al., 2017; He et al., 2020; Wei et al., 2021a). In addition, in the FINRISK 2002 cohort, *Parabacteroides* was identified as fractures risk factor (Grahnemo et al., 2023). Research supports the potential role of Parabacteroides in modulating host metabolism. For instance, Wang et al., demonstrated that *P. distasonis* influences bile acid metabolism and succinate production, which may contribute to reduced weight gain (Wang et al., 2019). However, further studies are needed to verify these findings and determine whether this association varies by age, gender, or specific microbial strains.

Growing evidence has shown that *Barnesiella*, *Oscillibacter*, and *Odoribacter* might be the key players in the progression of OS and OP in postmenopausal women. Kuo et al., reported that *Barnesiella*, and *Oscillibacter* are OP-associated (n = 21) in Taiwanese postmenopausal women (Kuo et al., 2023). A large-scale ethnic GWAS - microbiota study (n=34,024 individuals) reported that the *Barnesiella* genus was found to be a risk factor for BMD for individuals >60 years old (Wang et al., 2023b). In line, the genus *Oscillibacter* was increased in postmenopausal women with reduced BMD (Ma et al., 2024; Yan et al., 2024). However, contradictory results have been reported in Chinese postmenopausal women with OP (Dong et al., 2024). The presence of *Oscillibacter* in the gut is controversial. It may be positively influenced by the intake of soluble corn fiber, which is associated with enhanced calcium absorption (Jakeman et al., 2016). In addition, our results demonstrated a negative association between *Odoribacter* and BMD in postmenopausal women. This phenomenon was also observed in peri, and early postmenopausal women (Greenbaum et al., 2022). Lai et al., found *Odoribacter* sp*lanchnicus* was significantly higher in Chinese male/female with osteoporosis than in the normal bone density (Lai et al., 2024). A positive correlation between *Odoribacter* and the number of osteoclasts in femoral tissue in the OVX rat model suggests an essential role in bone resorption. Furthermore, a negative association with Foxp3 expression indicates a potential disruption of immune regulation and a proinflammatory intestinal environment (Zhu et al., 2024). However, contradictory results show that *Odoribacter* abundance was significantly lower in Chinese postmenopausal women with OP than in the normal BMD group (Liang et al., 2023). Although the data suggest that an increased abundance of *Oscillibacter* and *Odoribacter* may be linked to bone resorption and a proinflammatory gut environment, the contradictory results in different populations suggest that the relationship between GM and bone health is complex.

The *Sutterella* genus was also predominant among aged groups with low BMD. *Sutterella*, a member of the phylum Proteobacteria, may contribute to non-specific mucosal inflammation due to lipopolysaccharides acting as potent stimulators, potentially predisposing the host to a chronic inflammatory disease and its ability to degrade immunoglobulin A (IgA) (Hiippala et al., 2016). This degradation, mediated by IgA-specific serine endopeptidases, may facilitate bacterial invasion and persistence within host cells (Hiippala et al., 2016). A possible mechanism suggested that *Sutterella* may produce various metabolites such as short-chain fatty acids (SCFAs)or indirect effects through autoimmune-related bone density alterations (Tyagi et al., 2018; Cao et al., 2021). However, the precise role of this taxon in bone metabolism remains unclear and warrants further investigation.

The association between physiological parameters and gut microbial community is complex and not fully understood. Previous studies have reported that BMI and BMD alter gut microbial community structure. In our research, Bacteroidota members, including *Bacteroides*, *Parabacteroides*, *Coprobacter*, and *Odoribacter*, were negatively correlated with age and BMD measurements. This supports the hypothesis that these taxa could be responsible for changing the microbial diversity structure and may contribute to bone loss.

Multiple studies highlight short-chain fatty acids (SCFAs) as key regulators of bone composition (Han et al., 2024; Lucas et al., 2018). Our study identified SCFA producers, including *Butyricimonas*, and *Coprobacter* in the low-BMD group. In contrast, Qin et al., found *Butyricimonas* decrease in older adults with OP (n=88). Notably, the decline in butyrate-producing taxa (Lachnospiraceae and Ruminococcaceae) in our low-BMD group aligns with findings in other postmenopausal populations (Li et al., 2019a; Zaplana et al., 2024). Mechanistically, SCFAs (butyrate and propionate) directly suppress osteoclast differentiation by binding to receptors on osteoclast precursors, as demonstrated *in vitro* (Yan et al., 2018). However, while SCFAs inhibit osteoclast formation, they may lack efficacy against mature osteoclasts (Wu et al., 2023).

SCFAs may influence bone homeostasis providing a direct link between the gut microbiota and bone via immunomodulatory response (Tyagi et al., 2018; Li et al., 2019b). Propionate and butyrate act as histone deacetylase (HDAC) inhibitors, modulating NF-κB activity to exert anti-inflammatory effects. Butyrate enhances IL-10 production while suppressing proinflammatory cytokines (IL-12, TNF-α, IL-1β, and NO) and dampening inflammatory responses in intestinal macrophages (Liu et al., 2023). SCFAs promote the differentiation of naive T cells into Th1 and Th17 effector cells, likely through their HDAC-inhibiting effects. In this regard, certain *Bacteroides* and *Bifidobacterium* strains could contribute to IFNα production (Schirmer et al., 2016; López et al., 2010) by producing SCFAs and exopolysaccharides (Ai et al., 2021). For instance, *Bifidobacterium bifidum* strains enhance IL-17 secretion while suppressing IFNγ and TNFα, suggesting a possible Th17 profile. Conversely, *Bifidobacterium pseudocatenulatum* and *Bifidobacterium adolescentis* exhibit an opposite correlation with IFNα and TNFg (Schirmer et al., 2016), and *Bifidobacterium longum* exhibits protective effects by suppressing osteoclastogenesis and increasing bone mass density in experimental models (Sapra et al., 2022). Unexpectedly, higher *Bifidobacterium* abundance was associated with low BMD in our data, underscoring the need to elucidate strain-level mechanisms and contextual interactions within the gut-bone axis. Additionally, previous studies have reported that *Odoribacter* and *Barnesiella*, both Gram-negative bacteria, exhibit a negative correlation with TNFα production in response to LPS, stimulated ex vivo (Schirmer et al., 2016). This finding contrasts with our initial expectations and highlights the need for further environment analysis of bacterial interactions to clarify the bone-specific roles of immunomodulatory taxa and optimize SCFA-targeted interventions for skeletal health.

The Firmicutes/Bacteroidota (F/B) ratio has been associated with maintaining homeostasis, and changes in this ratio can lead to various pathologies. For example, increases in the abundance of specific Firmicutes or Bacteroidetes species lead to obesity (Clarke et al., 2014). In low BMD the results are contradictory. A meta-analysis (175 healthy controls vs. 177 OP patients) from five studies found no significant differences in the F/B ratio between groups (Akinsuyi and Roesch, 2023). In contrast, our study, observed a significantly decreased F/B ratio in the low BMD group. Similar data have been reported in Chinese postmenopausal women (>64 years) (Wang et al., 2017; Li et al., 2019a). These discrepancies may reflect variations in gut microbiota composition across different populations, size sample (Wang et al., 2017, 2022, 2023a; Das et al., 2019; He et al., 2020; Palacios-González et al., 2020; Xu et al., 2020; Qin et al., 2021; Rettedal et al., 2021), and methodological such as DNA extraction protocols, 16S rRNA gene targeted for amplification, and quality filtering parameters (e.g. Phred score). Maintaining a balanced intestinal ecosystem is crucial for normal body function, and many therapeutic strategies aim to achieve a suitable Firmicutes to Bacteroidetes (F/B) ratio.

The functional profiling of microbial communities reveals significant insights into metabolic pathways associated with bone health. In this study, the metabolic pathway analysis shows a significant depletion of valine, leucine, and methionine pathways in women with low BMD. Evidence suggests that the gut microbiota composition explains 19% of the variance of circulating, branched-chain amino acid (valine, leucine and isoleucine) (BCAA) concentrations (Dekkers et al., 2022). An enriched of Bacteroidaceae could explain the differences in serum BCAA concentrations between the groups, potentially contributing to bone loss. Similarly, a large population-based study (n=1776) reported negative correlations between leucine and valine concentrations and BMD in a Chinese population with OS and OP. The authors proposed that enrichment of ABPVCR consortium (*Actinobacillus* –*Bacteroides* – *Phascolarctobacterium* – *Veillonellaceae* – *Collinsella* - Ruminococcaceae) is associated with BCAAs degradation (Ling et al., 2021). Interestingly, Wang et al., identify fecal metabolites D-alanyl-D-alanine and serum serine-valine as inversely correlated with BMD (Wang et al., 2023a), confirming our results and reinforcing the link between microbial metabolism and bone health.

The potential benefits of BCAAs in bone maintenance have been extensively documented, highlighting their role in preserving bone integrity and supporting muscle-bone crosstalk (Zhao et al., 2018; Su et al., 2019; Grahnemo et al., 2023). Leucine, in particular, enhances mTOR-mediated protein synthesis, promoting osteoblast activity and bone formation while reducing bone resorption markers (Jennings et al., 2016). Meanwhile, valine has also been strongly correlated with bone health since it shares metabolic pathways with leucine, and has been proposed to exert a protective effect against fractures (Grahnemo et al., 2023). Additionally, dietary methionine supplementation improves hepatic steatosis, insulin resistance, inflammation, fibrosis, and bone health. Conversely, methionine deficiency has been linked to impairing osteoblast function, reduced bone formation and increased osteoclast activity (Ouattara et al., 2016). Although, these findings highlight emphasize the intricate relationship between microbial metabolism-AA production in skeletal integrity, experimental approaches (such as GM transplantation and metagenomic sequencing) are needed to identify key bacterial strains involved in amino acid metabolism and their role in low BMD.

On the other hand, estrogen deficiency is known to influence bone remodeling and alter lipid profiles; however, the application of plasma lipidomics in studying menopausal osteoporosis remains underexplored. In this study, we identified dysregulation in key lipid metabolism pathways, including those involved in alpha-linolenic acid, glycosaminoglycan, sphingolipid, and glycerolipid metabolism. It has been reported that plasma lipids and polar metabolites differ between women with normal and low BMD and are involved in several metabolic pathways, including sphingolipid and phospholipid metabolism, as well as fatty acid β-oxidation (Cabrera et al., 2018). Notably, two studies have linked specific sphingolipid species to low BMD in postmenopausal women, suggesting that sphingolipids may modulate bone metabolism via bone marrow-derived macrophages and could be attributed to estrogen deficiency (Cabrera et al., 2018; Lee et al., 2012). In contrast, other studies report inconsistent associations between triacylglycerol levels and hip BMD in this population (Cui et al., 2005; Brownbill and Ilich, 2006; Makovey et al., 2009), highlighting the need for further investigation.

Differences in the gut microbiome across populations are significant and driven by factors such as geography, ethnicity, diet, and lifestyle. Among these, diet plays a pivotal role in shaping the composition, function, and diversity of gut microbial communities. For instance, western diets rich in protein and fat are strongly associated with Bacteroides-dominant microbiomes, as observed in the US and Europe (Clemente-Suárez et al., 2023).

Regional variations in gut microbiota also have been documented (Gaulke and Sharpton, 2018). East Asian populations, particularly in China, Japan, and Taiwan, exhibit higher Bacteroides abundance, “BB-type”, compared to Southeast, Southern, and Central Asian populations (Nakayama et al., 2015; Gaulke and Sharpton, 2018; Gorvitovskaia et al., 2016). The “BB-type” gut microbiota is more prevalent in these regions, whereas the “P-type” microbiota, dominated by Prevotella, is more common in Southeast Asia (Gorvitovskaia et al., 2016).

Notably, older adults in both Asian and Mexican urban settings demonstrate higher protein and fat intake (Cho and Choi, 2021; Nabuco et al., 2018; López-Montoya et al., 2023) which could influence microbial composition and metabolic outcomes. However, while Asian gut microbiota research has identified specific bacterial taxa associated with BMD, the Mexican microbiome remains understudied in this context. These findings highlight the challenges in developing microbiome-based personalized medicine and underscore the necessity for region-specific research to account for such variations.

This study has several strengths. First, it comprehensively characterizes GM composition in a large cohort of Mexican postmenopausal women (n=535), offering valuable insights into this understudied demographic. Second, it identifies specific GM taxa associated with BMD status and explores their metabolic pathways, particularly those involved in AA metabolism, which may influence BMD. However, the study also has present limitations. First, the regulation of circulating AA concentrations is a complex physiological process influenced by multiple factors, including dietary intake, protein turnover, the novo synthesis and metabolic clearance. Thus, the specific contribution of gut microbiota to AA metabolism remains unclear and requires further validation.

Second, GM composition is influenced by diet, age, geography, and host health status. For instance, diet can account for up to 20% of the variation in GM composition, which also fluctuates seasonally (Farhat et al., 2023). Notably, phytoestrogen intake has been shown to enhance the diversity and abundance of beneficial gut bacteria, thereby modulating their effects on sex hormones (Gyriki et al., 2025). Although this variable was not included in the questionnaire, its potential impact warrants consideration in future research. Third, GM undergoes age-related changes, including reduced diversity of commensal bacteria and increased in pathogenic species, which may contribute to disease susceptibility. Another key factor in gut dysbiosis is antibiotic use, which has been shown to disrupt sex hormone metabolism, bile acid metabolism, and the synthesis of fats and vitamins (e.g., vitamin K)— (LeBlanc et al., 2013; Collins et al., 2023) all of which may influence bone health regulation.

Four, several studies have determined the impact of single-nucleotide variants (SNVs) related to amino acid metabolism (Imaizumi et al., 2019; Lares-Villaseñor et al., 2024) on serum concentrations. However, the bidirectional interactions between host genetics, GM, and serum amino acids remain underexplored. Future studies should investigate whether these genetic variants exhibit a causal relationship with bone loss over time and elucidate the underlying mechanisms, including potential mediation by microbial metabolites or host-microbe metabolic crosstalk.

Lastly, while probiotics and prebiotics have demonstrated osteoprotective effects in other studies (Sapra et al., 2021; Farhat et al., 2023), their role in this specific population remains unexplored. Although this study partially accounts for some of these factors, the heterogeneity in bacterial associations with bone loss across the literature underscores the need for further research to fully elucidate these complex interactions.

## 5 Conclusions

The results showed that postmenopausal women with low bone mineral density (BMD) experience significant changes in their gut microbiota and serum metabolites. These changes are closely correlated with BMD measurements, similar to the Caucasian and Asian populations. This correlation offers potential insights into the mechanisms behind the low BMD and may serve as an early diagnostic indicator. This study could pave the way for new interventions to improve bone health in Mexican postmenopausal women.

## Data Availability

The datasets presented in this study can be found in online repositories. The names of the repository/repositories and accession number(s) can be found in the article/**Supplementary Material**.
