# Supplementary material for: Gut microbiota changes in postmenopausal women with low bone density linked to serum amino acid metabolism
Source: Front Cell Infect Microbiol. 2025 Jul 9;15:1627519. doi: 10.3389/fcimb.2025.1627519 (PMC12283679; doi:10.3389/fcimb.2025.1627519)
Supplement: Supplementary file 1 [file Table1.docx]

Supplementary Material


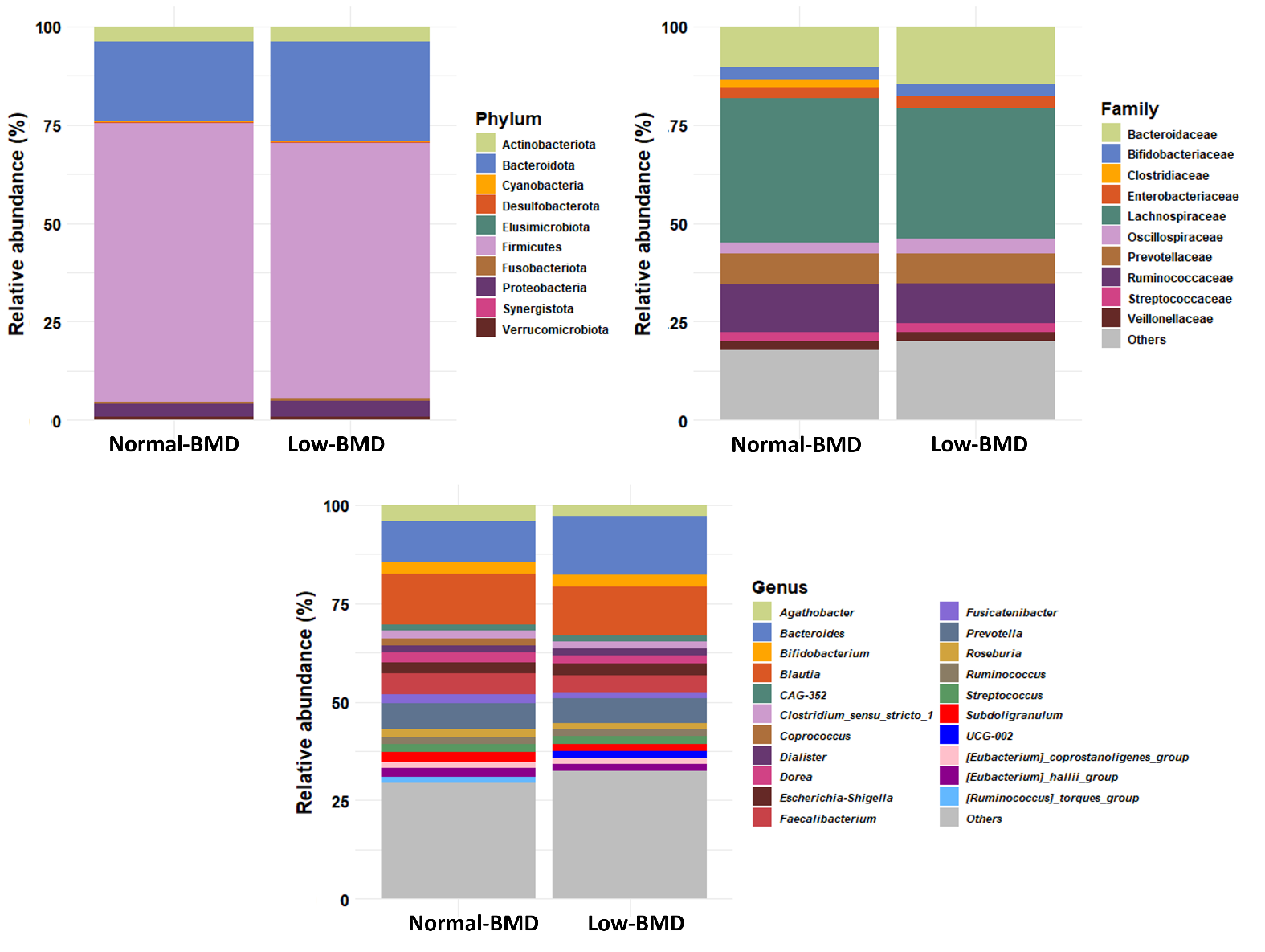


**Supplementary Figure 1.** Supplementary Figure 1 Relative abundance distribution of gut microbiota in low-BMD and normal-BMD groups. Bacterial composition at (A) Phylum level, (B) Family level, (C) Genus level.


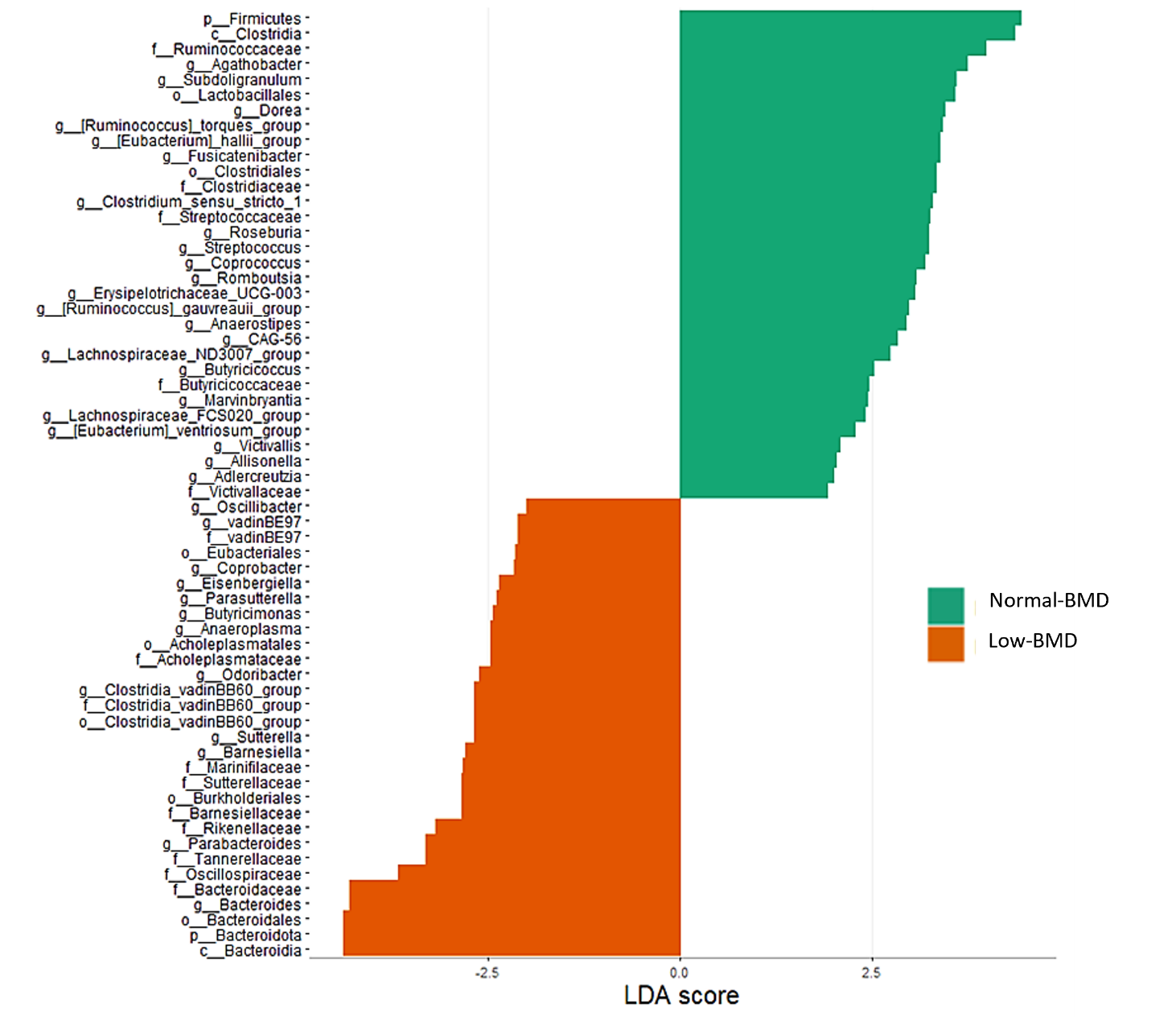


**Supplementary Figure 2** Taxonomic gut microbiota differences identified by LEfSe analysis between normal-BMD women (green) and low-BMD women (orange). (p) phylum; (c) class; (o) order; (f) family; (g) genus. Taxa with LDA score > 1.5 and *p* < 0.05 are shown.


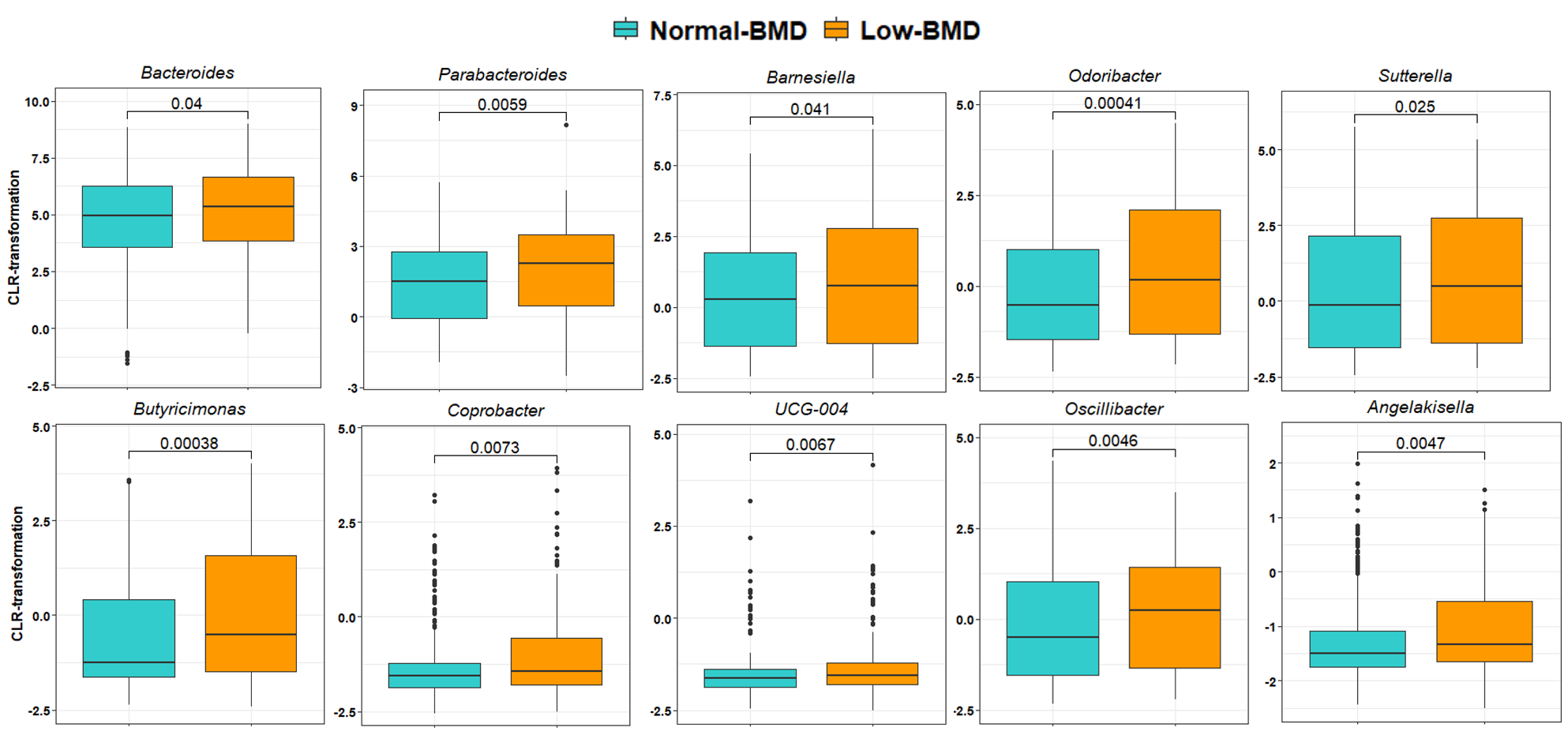


**Supplementary Figure 3** Boxplots representation of significant genera. The differences between groups were performed using Wilcoxon sign rank test.


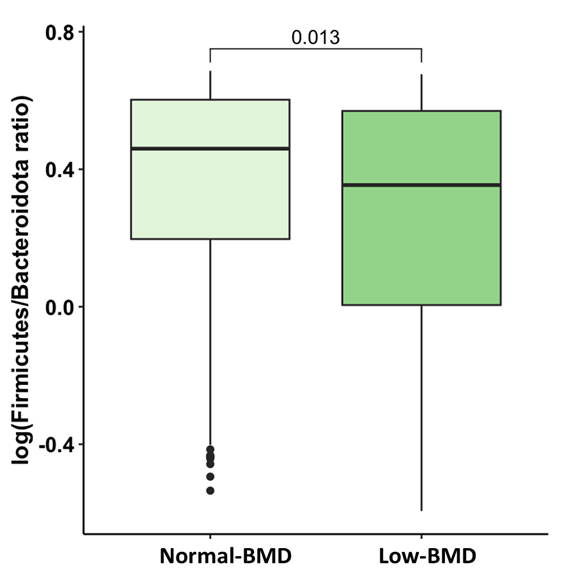


**Supplementary Figure 4** Comparison of Firmicutes/Bacteroidota ratio between the low-BMD and normal-BMD groups. Values were log10-transformed.


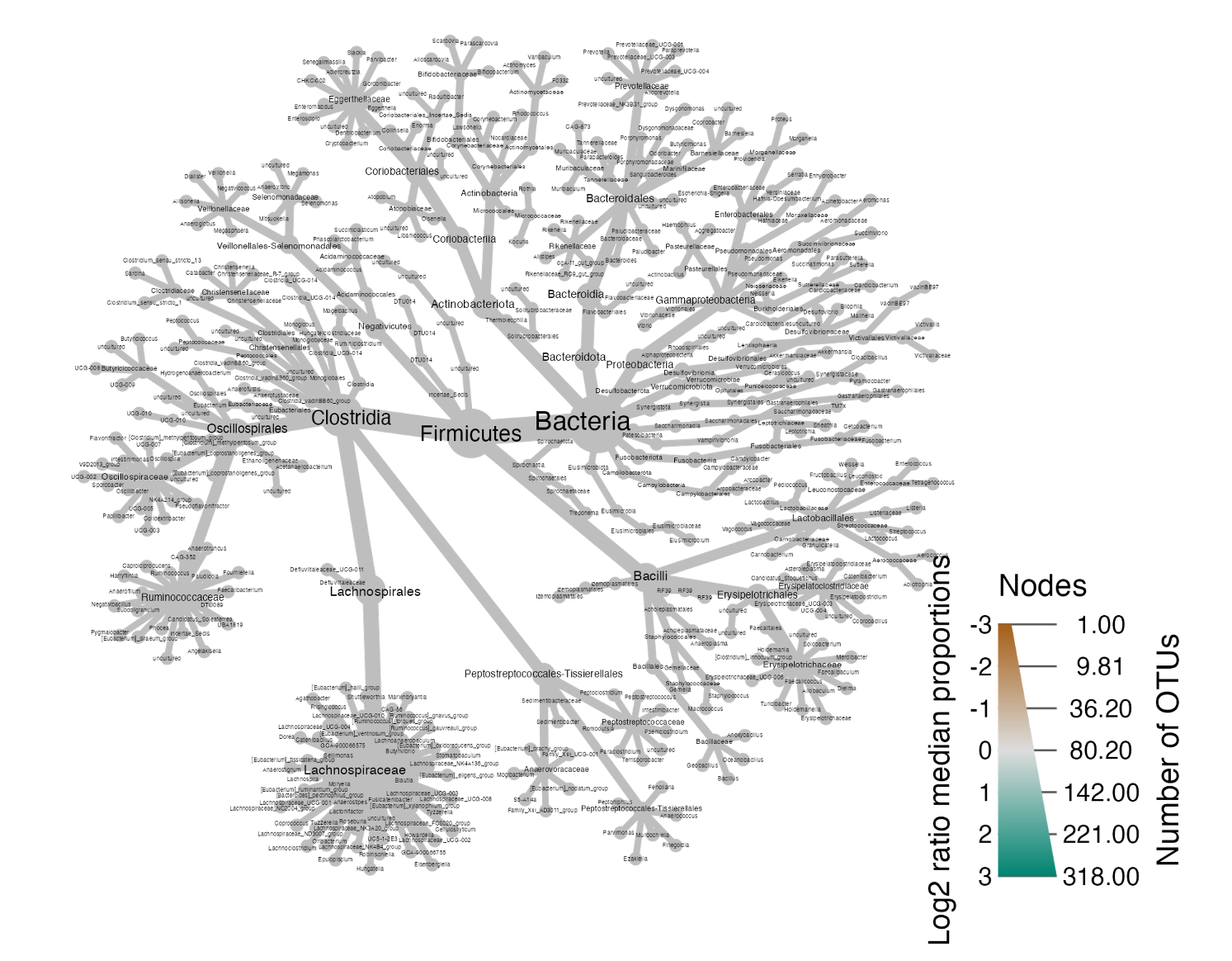


**Supplementary Figure 5**. Heat tree base of for pair-wise comparison, divided by BMD-age.


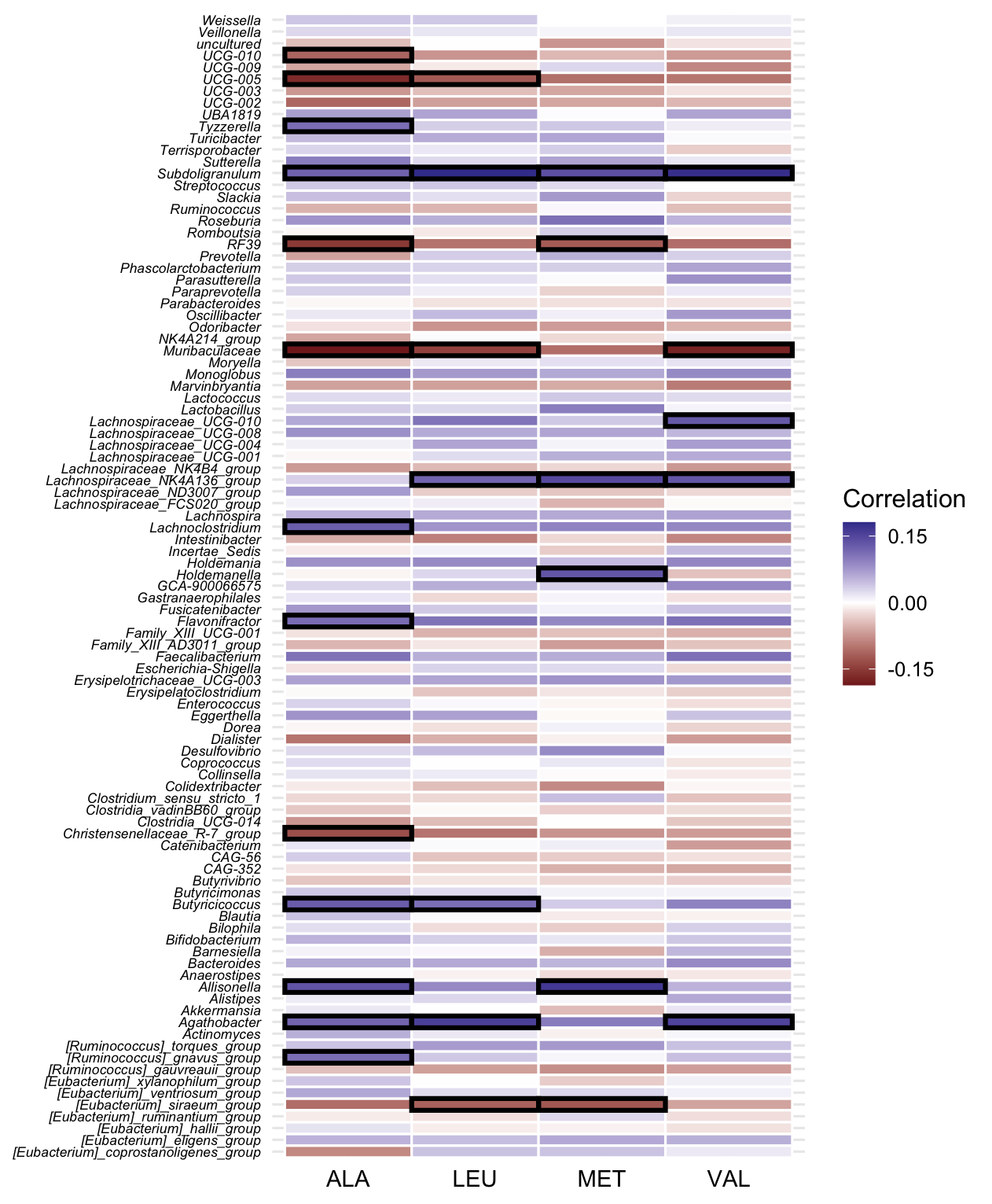


**Supplementary Figure 6.** Heatmap of partial Spearman’s rank correlation analysis between bacterial genera and serum amino acid concentration. Black square frame represents a significant correlation (p < 0.05). Red squares represent negative correlations and blue squares represent positive correlations. *Subdoligranulum* abundance was positively correlated with alanine, leucine, methionine, and valine. UCG-005 was negatively correlated with alanine, and leucine. ALA: alanine, LEU: leucine, MET : methionine, VAL : valine.


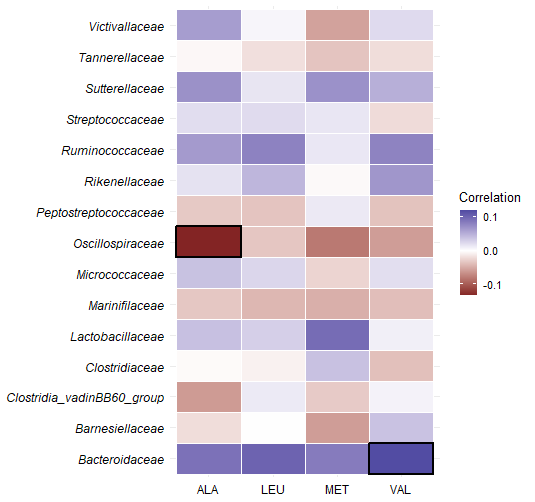


**Supplementary Figure 7**. Heatmap of partial Spearman’s rank correlation analysis between bacterial family and serum amino acid concentration. Black square frame represents a significant correlation (p < 0.05). Red squares represent negative correlations, and blue squares represent positive correlations. Oscillospiraceae family abundance was negatively correlated with valine, and Bacteroidaceae family abundance was positively correlated with valine. ALA: alanine, LEU: leucine, MET : methionine, VAL : valine.
